# RECENT ADVANCES IN ASSESSING AGE DIFFERENCES IN AFFECT DYNAMICS

**DOI:** 10.1093/geroni/igad104.0979

**Published:** 2023-12-21

**Authors:** Maria Wirth, Mai Mikkelsen, Susan Charles

## Abstract

Affective experiences are inherently dynamic and short-term changes in affect offer important insights into well-being. Changes in the processes underlying affect dynamics, namely, affect reactivity and regulation, are often invoked as explanations for age-related stability or enhancement of well-being. However, previous evidence for age differences in affect dynamics is inconsistent. In this symposium, we leverage theory-based approaches and cutting-edge statistical tools to gain a deeper understanding of how individuals manage daily affect. Wirth and colleagues investigated age differences in affect reactivity and regulation, using a formalized theoretical approach to affect dynamics. Their results indicated complex, multi-directional age differences in reactivity and regulation. Neupert and Graham focused on the idea that perception of time remaining in life plays a key role in age differences in affect dynamics. Feeling closer to death was related to lower positive and higher negative affect. These relations were particularly pronounced in older adults. Wolfe and colleagues investigated age-differential associations between cognitive capacities and affect regulation preferences. Their results indicated that cognitive capacities affected regulation preferences mostly in middle-aged rather than older adults. Mikkelsen and colleagues related affect regulation patterns to well-being in an age-diverse sample. Their findings indicated that older adults exhibit less variation in regulation strategy use and that regulation effectiveness was less important for well-being in older adults. The discussion will center on the value of considering age-related similarities and differences in affect dynamics, age-relevant factors for successful emotion regulation, and the contexts that might influence affect dynamic processes.

